# Technology-enhanced learning: a role for video animation

**DOI:** 10.1038/s41415-020-2588-1

**Published:** 2021-01-22

**Authors:** Bernd Stadlinger, Søren Jepsen, Iain Chapple, Mariano Sanz, Hendrik Terheyden

**Affiliations:** 1 Clinic of Cranio-Maxillofacial and Oral Surgery, Centre for Dental Medicine, University of Zurich, Plattenstrasse 11, CH-8032 Zurich, Switzerland; 2 Department of Periodontology, Operative and Preventive Dentistry, University of Bonn, Welschnonnenstraße 17, D-53111 Bonn, Germany; 3 School of Dentistry, Periodontal Research Group, University of Birmingham, Birmingham, B5 7EG, UK; 4 Facultad de Odontología, Universidad Compludense, Plaza Ramón y Cajal s/n (Ciudad Universitaria), 28040 Madrid, Spain; 5 Department of Oral & Maxillofacial Surgery, Red Cross Hospital, Hansteinstraße 29, D-34121 Kassel, Germany; Department of Oral & Maxillofacial Surgery, Lecturer, University Hospital Schleswig-Holstein, Arnold-Heller-Straße 3, D-24105 Kiel, Germany

## Abstract

The last 20 years has seen a shift in medical education from printed analogue formats of knowledge transfer to digital knowledge transfer via media platforms and virtual learning environments. Traditional university medical teaching was characterised by lectures and printed textbooks, which to a degree still have an important role to play in knowledge acquisition, but which in isolation do not engage the modern learner, who has become reliant on digital platforms and 'soundbite' learning. Recently, however, traditional methods of teaching and learning have been augmented by, and indeed sometimes replaced by, the alternative learning methods such as: problem-based learning; a greater integration of basic science and clinical considerations; smaller teaching groups; the 'flipped classroom' concept; and various technological tools which promote an interactive learning style. The aim of these new teaching methods is to overcome the well-documented limitations of traditional lectures and printed material in the transfer of knowledge from expert to student, by better engaging the minds of more visual learners and encouraging the use of diverse resources for lifelong learning. In this commentary paper, we share the concept of video animation as an additional educational tool, and one that can help to integrate molecular, cellular and clinical processes that underpin our understanding of biology and pathology in modern education. Importantly, while they can provide focused and attractive formats for 'soundbite' learning, their aim as a tool within the broader educational toolbox is to direct the interested reader towards more traditional formats of learning, which permit a deeper dive into a particular field or concept. In this manner, carefully constructed video animations can serve to provide a broad overview of a particular field or concept and to facilitate deeper learning when desired by the student.

## Key points

- Teaching the knowledge explosion is a challenge.
- Video animation plays a future role in teaching complex biological concepts and processes.
- Video animation helps in educating professionals and the public.

## The role of media and film in medical education

Various media have been employed to reach this goal. In the twentieth century, films/movies became increasingly used as teaching media. As early as 1913, Thomas Edison is said to have declared that books would become obsolete in schools. Although this prediction has not been fully realised, films have indeed played an increasing role in modern education. Filming real action sequences can allow material to be presented in a consistent manner and can introduce elements of entertainment to engage and stimulate the audience, but overall, movies as educational tools still have similar limitations to lectures, in that the audience is passive and the movie is limited to real events that can be filmed. More recently, video animation has allowed greater imagination in the presentation of educational material, and over the past ten years, this has been enriched by means of augmented and virtual reality. For many movie audiences, the film *Avatar* in 2009 provided an early indication of the potential offered by such technologies. Developments in digital technology aim to make the consumer an increasingly integral part of the digital environment. In this context, the term 'digitality' is used in humanities and may replace the term digitalisation in the future. Digitality describes a holistic virtual experience. A particular challenge in medicine is the transition between three levels of magnification: the macroscopic, the microscopic and the molecular-biochemical level. All three levels provide explanations for biological and pathological processes within organs, tissues and their constituent cells, as well as the processes of disease resolution and healing. Modern virtual three-dimensional (3D) modelling can help visualise these three levels as connected processes, with time as the fourth dimension.

## The challenge of teaching the knowledge explosion

At the same time, medical scientific knowledge has increased rapidly and in dentistry knowledge now doubles every five years,^1^ a pace that has surpassed the adaptation of teaching methods. Thousands of new medical publications appear each year and it is impossible for a single individual to follow all developments in medical research. There is therefore a need for novel formats to provide an overview of subjects and disciplines, providing students and practitioners with accessible and attractive means to understand research topics in a reasonable timeframe. In recent years, research in the field of cell biology has provided deep insights into communication pathways between cells and how those translate to visible clinical outcomes. To teach cause-and-effect relationships in complex diseases, simulations can be of value, as they can be in many areas of education. It remains unclear to what extent methods of simulation can be used to impart knowledge of cell biology in medical teaching, but modern students appear to appreciate and support their use, as evidenced by website viewing statistics. This is of special interest for the mouth as an organ, where highly complex cell processes take place in a specialised environment, where a multitude of prokaryotic and eukaryotic cell types manifest themselves simultaneously, unlike any other kind of organ.

## Video animation and its future role in teaching complex biological concepts and processes

**Fig. 1:**
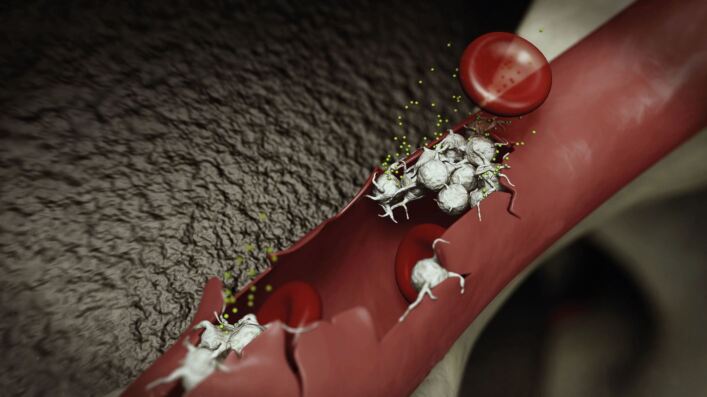
Platelet aggregation at a ruptured vessel, following implant insertion. Implant surface is shown in the background.^2^^,^^3^ (Courtesy of Quintessence Publishing.)

It is important that students and practitioners understand the complex interplay between the cascade of underpinning biological processes and clinical manifestations of those interactions. This opens the door for computer-animated films. A clinical course of treatment can be simulated by means of a film where cellular and even molecular effects can be visualised in a way that has both clarity and the ability to engage all three levels of human tissue structure and function as a continuum. To this end, modern techniques of video animation were used for a computer-animated film on the osseointegration of dental implants, complications of those implants and their management, so that students could gain a visual understanding of the interaction between biomaterials and tissues, in a way that could easily be explained in words or by static diagrams, or via a traditional film. A major advantage of a computer-animated film is the possibility of describing not only interactions between cells and biomaterials at single points in time (for example, osteoblasts on implant surfaces) but also the interactions along a time axis (the fourth dimension). In the case of osseointegration, this means that the course of the phases of wound healing can be described and visualised via activation and inhibition mechanisms of specific cell types (Figure 1).^2^^,^^3^

**Fig. 2:**
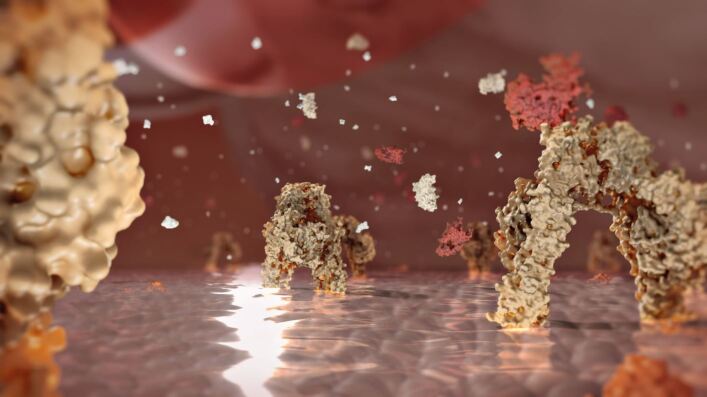
Insulin receptor.^3^^,^^4^ (Courtesy of Quintessence Publishing.)

Along with the simulation of a clinical course of treatment, a computer-animated film can be used to illustrate the progression of a disease. One example is the development of periodontitis and its possible association with systemic diseases such as artherogenic cardiovascular disease (arteriosclerosis) or diabetes mellitus (Figure 2).^3^^,^^4^ From an imaging perspective, the animated film can move seamlessly from the macroscopic perspective of the practitioner, through the microscopic perspective of vessels and cells, and into the molecular-biochemical perspective of proteins, lipids and DNA. A continuous film needs to fade in and out between these different perspectives without the viewer losing their orientation. Using these techniques, underlying pathologies of tissues can be visualised; for example, a clinical swelling will be explained by an underlying shift of fluid from the intravascular to the extravascular space.

Such models allow the simulation of clinical situations or treatment pathways with the underlying 'invisible' biological processes that underpin them, creating what is essentially a spatial model. A challenge in such projects is the balancing act between the need to present the latest medical knowledge and the risk of over-complicating the message. The information presented and the images employed need to be focused on the key messages to be conveyed, so that audience attention is maintained. This requires a panel of experts to develop, based upon careful appraisal of the current literature, which messages need to be imparted in each scenario.

To date, our team has produced six computer-animated films under the title 'Cell-to-cell communication'. The initial idea for this project came during a PowerPoint presentation of one of the authors on wound healing. A series of slides showed the interaction of cells and their mediators in the wound healing cascade. One member of the Quintessence team approached the speaker after the presentation suggesting that our brain is somehow not prepared to understand and memorise the complex molecular interactions presented. At that point, the idea was born to deliver this knowledge via a film in a 4D-multidimensional format. This can be referred to as enlarged knowledge space, allowing the observer to visualise cause-and-effect pathways over time, focusing on the main messages. Such a knowledge space can be realised via a computer-animated film. 

It is important not to overload the film with too many details, but to focus on key messages that are integrated via a storyboard. Such a storyboard needs creative drama and actors. In our series, we chose different cell types as the principal actors and their mediators as the vehicle through which they express themselves. The creative design is a clinical story; for example, the previously described osseointegration of a dental implant being explained by the four phases of wound healing. Therefore, each film has a clinical message linked to a basic science topic. The two films 'Osseointegration'^2^ and 'Guided bone regeneration'^5^ focus on bone biology, vascularisation and osteoimmunology. The two films 'Inflammatory reactions'^6^ and 'Periodontal regeneration'^7^ focus on the immune system, tooth development and regeneration. The film 'Peri-implantitis and its prevention'^8^ addresses the oral microbiome in symbiosis and dysbiosis, its interaction within the human immune system, and the consequences for the peri-implant soft and hard tissues. Finally, the film 'Oral and systemic health' bridges the gap from the oral cavity to general medicine, and shows the relationship between oral and systemic deseases like arteriosclerosis and diabetes mellitus.^4^ As the density of knowledge conveyed in a film can easily exceed the optimum, we decided to write an accompanying descriptive narrative paper for each film.^9^^,^^10^^,^^11^^,^^12^^,^^13^ In one case, an editorial was written instead.^14^ As such, those who are interested in a deeper dive into greater detail can refer to the accompanying papers.

## Educating specialists, students and the public via video animations

A challenge was to reach an audience of specialists as well as the public. For this reason, each film has two narrated versions - an expert and a public version. While both audiences see the same images, the text differs in terminology; for example, talking about bone-building cells instead of osteoblasts. As such, the public is being aligned to professional messaging in a visually attractive manner (the film series can be found here: In order to embed each film project into the scientific community, an international expert board of university-based clinicians and researchers ensures that the film is based on the latest scientific knowledge and international consensus views. These experts are selected based on their active research on the subject of the film project, in order to gain a broad, international appeal. Due to the international distribution of the authors and the advisory board, every film is produced in English, German and up to ten different languages. In order to use the films for teaching, the storyboard, the review paper and a multiple-choice test are integrated into a film booklet. All films can be downloaded by any university free of charge. To cover the cost of the production, a company partnership exists for each film.

To the best of our knowledge, there are no comparable educational tools currently available in dental medicine. The 15-20-minute-long computer-animated films can be integrated into lectures and subjectively appear to gain high acceptance, as twenty-first-century students embrace digital technologies in all aspects of their lives. The films do not replace a lecture but can be used as an additional teaching tool or to augment lectures.

One limitation is that the intensive scientific knowledge being embedded in a 15-minute film can overburden the observer. Therefore, it is important to only discuss those cells and messengers that are shown in the film images and to design cellular pathways in the storyboard, which can be clearly followed and understood. A full story with a clearly defined starting and finishing point, based on a clinical process, is the key element for an understandable and impactful film. The general aim is to improve the understanding of biology and pathology in dental medicine.

## Future considerations

Video animation, mixed media, and augmented and virtual reality are emerging techniques that are gaining an important place in medical and dental education and clinical settings. Various studies have analysed their impact on education and clinical training.^15^^,^^16^^,^^17^^,^^18^ A next step after the simulation of a course of disease or treatment is the establishment of interactive simulation models. A common feature of biological systems is that there is seldom a monocausal influence, but multiple biological systems, such as activators and inhibitors of a process, which balance each other in a complex interplay. The balance between pro- and anti-inflammatory conditions in a healing wound is an example of this. Complex systems are difficult to understand for the human brain and a computer simulation can help to visualise that complexity, while at the same time demonstrating the influence of a single factor on the entire system. These concepts move beyond the linear presentation of a clinical treatment procedure to confront the student or practitioner with expected sequelae and unexpected events, in a manner analogous to a flight simulator in aviation training. Such simulators are already in use in various medical specialties like ophthalmology, gynaecology and laparoscopic surgery.^19^^,^^20^^,^^21^ Next to their advantage as teaching aids, they can be used as a tool for quality control before the performance of a specific procedure. These simulations can be enriched by means of augmented or virtual reality.^22^ At a time when virtual reality is penetrating many areas of life - such as computer games, the automobile industry, architectural simulations and many more - it is very interesting to investigate the possibilities of using this approach for medical purposes. In summary, computer-aided virtual reality simulations of complex biological concepts that have meaningful clinical implications (and which employ photorealistic, computer-animated images in a modern and accessible visual media format) have an important role to play in twenty-first-century medical education. Medical guidelines can be integrated into such simulations in order to transform the factual knowledge conveyed by the video into knowledge of processes and their outcomes. This represents a format and platform that the current generation of learners understand and connect with, and can augment traditional formats for medical education.
